# Editorial: Neurobiological Systems Underlying Reward and Emotions in Social Settings

**DOI:** 10.3389/fpsyt.2021.644672

**Published:** 2021-02-05

**Authors:** Monika Eckstein, Anna-Lena Zietlow, Martin Fungisai Gerchen, Jonathan Levy

**Affiliations:** ^1^Institute of Medical Psychology, Heidelberg University Hospital, Heidelberg, Germany; ^2^Ruprecht-Karls University Heidelberg, Heidelberg, Germany; ^3^Department of Psychology, School of Social Sciences, University of Mannheim, Mannheim, Germany; ^4^Research Group Biological Psychology, Department of Clinical Psychology, Central Institute of Mental Health, Heidelberg University/Medical Faculty Mannheim, Mannheim, Germany; ^5^Department of Psychology, Heidelberg University, Heidelberg, Germany; ^6^Bernstein Center for Computational Neuroscience Heidelberg/Mannheim, Mannheim, Germany; ^7^Department of Neuroscience and Biomedical Engineering, Aalto University, Espoo, Finland; ^8^Interdisciplinary Center Herzliya (IDC), Baruch Ivcher School of Psychology, Herzliya, Israel

**Keywords:** social interaction, emotions, multidisciplinarity, mental health, neuro-phenomenology, stress, reward, empathy

Emotions and reward are central to almost every aspect of human social life. The goal of this Research Topic was to collect new relevant research reports and theoretical frameworks on the neurobiological and psychological mechanisms underlying emotions and reward in different social settings, from the perspectives of neuroscience, biology, neurology, social medicine, philosophy, and psychology.

The human brain can be characterized as an inherently social organ; permanently adapting its function to the social context and constantly influenced by social interaction. In fact, it has been argued that the information processing capacity necessary for representing the complex social relationships in social groups was one of the driving factors in the evolution of the large primate and human cortex (1, 2). For an individual, being integrated in close social relationships has considerable consequences for physical and mental health and even for survival. This impact can even exceed the benefits of physical activity or absenteeism from alcohol (3). Further, most of the most frequent and debilitating mental disorders, like depression, anxiety disorders, or personality disorders, are characterized by profound deficits in social interactions, social cognition and emotion regulation as well as disturbances in social brain networks (4). The complementary use of new experimental paradigms and technologies in research (e.g., neuroimaging or virtual-reality) in the fields of psychiatry, neurology, neuroendocrinology, or phenomenology is necessary for a nuanced investigation of the mechanistic bases of social phenomena and might stimulate innovative multidisciplinary-based diagnostic and treatment strategies.

For a successful translation of basic science to implementation, research needs to integrate empirical experiments, clinical investigations, and theoretical models. We have covered all those three stages of research in our collection of 14 articles.

First, neural mechanisms of social cognition, emotions, and stress processing have been investigated in basic research with healthy subjects: Using functional magnetic resonance imaging (fMRI) and an empathic mirroring task, Ho et al. investigated an intervention designed for reducing parental stress. They found that the intervention's stress -reducing effect was mediated via selective neural responses and information trafficking patterns. Two other studies used electroencephalography (EEG) and emotional faces as stimuli; Yang et al. reported neural events underlying automatic and regulatory patterns of emotion appraisal, whereas Liu et al. found an interesting relationship between frontal EEG alpha asymmetry and individual differences in the processing of congruent and incongruent fearful faces. Similarly, but using diffusion tensor imaging (DTI) and fMRI, Jung and Kim looked at individual differences in the tendency to compare oneself with others, and found that these were predicted by different patterns of functional and structural brain connectivity. For the very relevant but understudied research field of olfaction, behavioral and fMRI evidence shows that social signals are transported with smells. Schäfer et al. demonstrate that mothers are able to detect the developmental state of children by smelling their body odors, especially when they have own children of similar age. Smelling odors of stressed persons leads to an increased activation of the Amygdala and related networks in healthy subjects that have a history of childhood trauma, as reported by Maier et al. This hypersensitivity to stress signals can be dampened by oxytocin.

Second, with a focus toward psychiatric patients, further clinical research is reported: An fMRI study on reward and affect by Soelch et al. found that increased reward-related neural activation during stress exposure was associated with positive affect in the daily life of young adults with a family history of depression. For females suffering from acute major depression, Warth et al., could show that an instructed positive interaction with their romantic partners results in higher stress levels, as assessed with cortisol on the one hand, but also improved relationship quality on the other hand. Therefore, adding to the evidence that the interaction of reward and stress is modulated in affective disorders. Likewise, in a single-subject study on a patient with acquired damage of bilateral amygdalae, Piretti et al. integrate the neural level with the subjective experience of emotions, thereby pointing out the interaction between this neural substrate and subjective shame during social norm violations. Kroczek et al. investigated interpersonal distance in social interaction using a novel paradigm in virtual reality to study social anxiety behavior of avoidance in real-life settings, and by integrating subjective experience, behavior, and physiology. For patients suffering from Borderline Personality Disorder, Schneider et al., could show a beneficial effect of applying the neuromodulator oxytocin for behavioral hypersensitivity/avoidance toward threatening facial stimuli. These findings nicely complement the above mentioned Maier et al.'s study of oxytocin's dampening effect to stress hypersensitivity in healthy controls with a history of childhood maltreatment.

At the third stage, in order to draw joint conclusions, review, and theoretical articles aimed to combine the heterogenous literature and integrate multiple perspectives; in these reviews, we particularly focused on integrating subjective and neurobiological proxies of emotions, stress and reward during contexts of social interaction. Eckstein et al. reviewed the current state of research on the role of social and non-social (robotic) touch for stress-relief from a medical as well as technical view, taking into account subjective experience and objective physiology. Matyjek et al. summarized multiple dimensions of social and non-social rewards, such as duration, familiarity, and source in a model in order to allow a differentiated description and recommendations for experimental comparisons. Levy and Bader follow on the empirical data above on subjective experience of emotions, empathy, and the integration of these experiences with neural data. They do so by providing a novel neuro-phenomenological framework (i.e., integrating neuroscience and subjective experience) on empathy, thereby extending dichotomous accounts and bringing forward an ecologically valid approach to real-life empathic encounters, while reporting empirical supporting evidence from magnetoencephalography (MEG) and other neuroimaging studies.

Taken together, the collection of studies in this research topic provides a multi-disciplinary outlook on emotions, reward, and social interactions by accumulating evidence from numerous neuroimaging techniques (MEG, EEG, fMRI, DTI), phenomenology, hormones, olfaction, virtual-reality, interventions, behavioral paradigms, and patient studies (Figure 1). The heterogeneity of these studies reflects the multiplicity of scientific approaches to study the complex processes and mechanisms involved in emotions and reward in social settings. This is particularly relevant at a time when (non-digital) social interaction is being reduced due to the Covid-19 pandemic, while providing an outlook on the involvement of multiple levels during social interactions. Thus, the research topic can motivate researchers to reproduce these findings and to test new hypotheses. For instance, what are the differences in mechanisms implicated during digital vs. non-digital social interaction? Does it influence interpersonal empathy? Does it implicate more stress and less reward? Finally, this topic can pave the road toward designing innovative interventions targeting the different levels of social interaction as outlined here (Figure 1).

**Figure 1:**
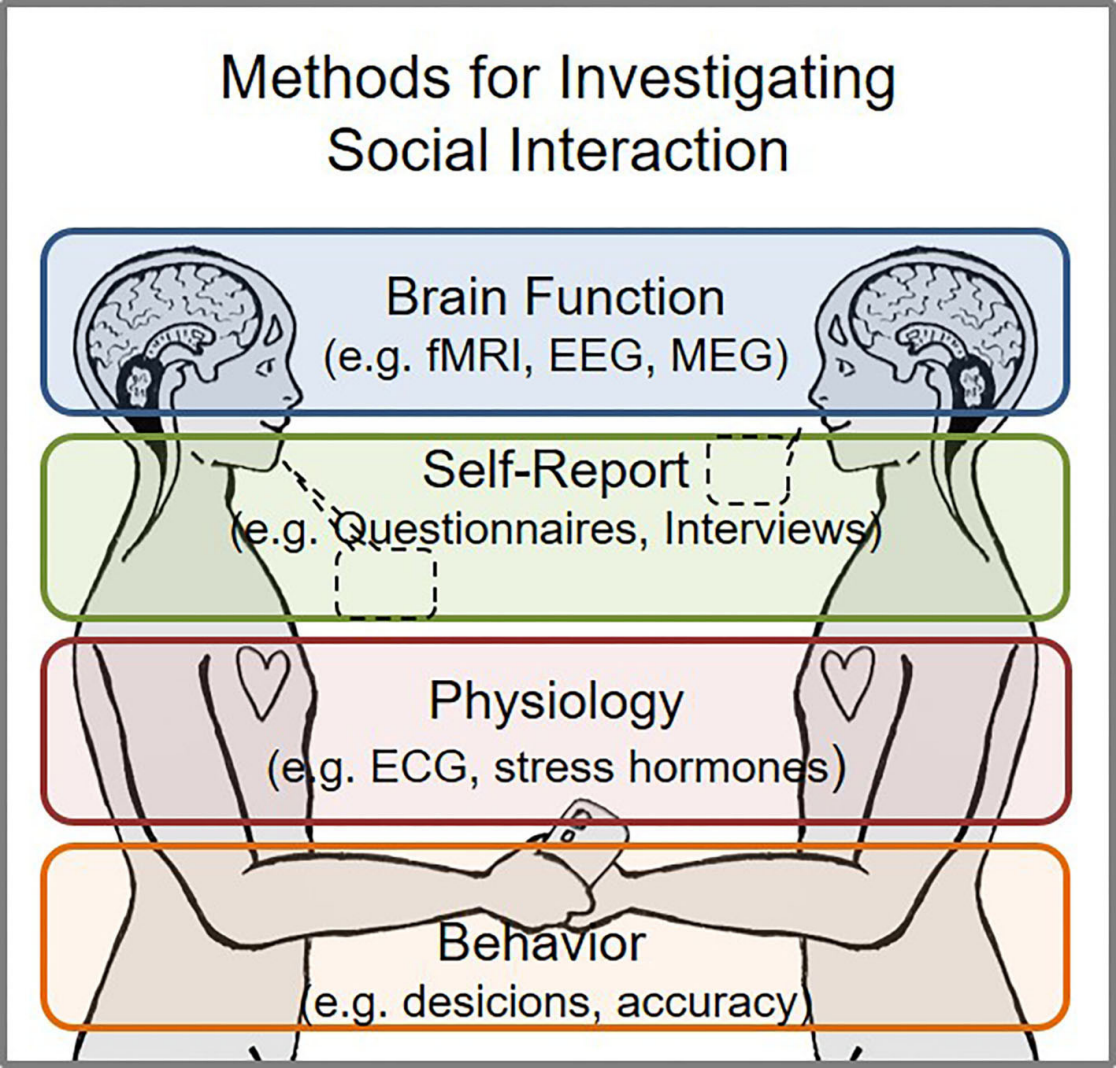
Overview on multi-method perspectives for investigating social interaction.

## Author Contributions

ME and JL took the lead in writing this editorial while all authors contributed to finalizing it.

## Conflict of Interest

The authors declare that the research was conducted in the absence of any commercial or financial relationships that could be construed as a potential conflict of interest.
